# Distal nucleotides affect the rate of stop codon read‐through

**DOI:** 10.15302/J-QB-022-0298

**Published:** 2023-03-01

**Authors:** Luciana I. Escobar, Andres M. Alonso, Jorge R. Ronderos, Luis Diambra

**Affiliations:** ^1^ CREG Universidad Nacional de La Plata‐CONICET La Plata CP 1900 Argentina; ^2^ INTech Universidad Nacional de San Martin Chascomus CP 7130 Argentina; ^3^ FCNyM Universidad Nacional de La Plata La Plata CP 1900 Argentina

**Keywords:** translational readthrough, stop codons, translational termination, ribosomal density profiles, nucleotide usage frequency

## Abstract

**Background:**

A key step in gene expression is the recognition of the stop codon to terminate translation at the correct position. However, it has been observed that ribosomes can misinterpret the stop codon and continue the translation in the 3′UTR region. This phenomenon is called stop codon read‐through (SCR). It has been suggested that these events would occur on a programmed basis, but the underlying mechanisms are still not well understood.

**Methods:**

Here, we present a strategy for the comprehensive identification of SCR events in the *Drosophila melanogaster* transcriptome by evaluating the ribosomal density profiles. The associated ribosomal leak rate was estimated for every event identified. A statistical characterization of the frequency of nucleotide use in the proximal region to the stop codon in the sequences associated to SCR events was performed.

**Results:**

The results show that the nucleotide usage pattern in transcripts with the UGA codon is different from the pattern for those transcripts ending in the UAA codon, suggesting the existence of at least two mechanisms that could alter the translational termination process. Furthermore, a linear regression models for each of the three stop codons was developed, and we show that the models using the nucleotides at informative positions outperforms those models that consider the entire sequence context to the stop codon.

**Conclusions:**

We report that distal nucleotides can affect the SCR rate in a stop‐codon dependent manner.

## Abstract 2

**Author summary:** Sometimes ribosomes can misinterpret the stop codon and continue the translation to produce an extended protein. These events can occur by chance, as well as, by a programmed mechanism. However, the basis of this mechanism is still not known. In this paper, we report that the codon usage bias, at the end of the transcripts with UAA stop‐codon, are a key determinant of the stop codon read‐through. The non‐optimal codon usage suggests that the canonical interpretation of the UAA codons might require ribosomal pause at the end of the coding region of the transcript.

## INTRODUCTION

Protein synthesis is completed in both, prokaryotes and eukaryotes, when ribosomes encounter one of the three termination codons (UAA, UAG and UGA). This final step involves the recognition of a termination codon, and the release of the completed polypeptide from the last tRNA, followed by the dissociation of ribosomes from mRNA. In eukaryotes, the stop codon recognition is based on the mRNA compaction driven by the interaction of eRF1 with the nucleotide A1825 of 18S rRNA [1]. There exist also mechanisms that can lead ribosomes to continue translation beyond the first termination codon, resulting in a fraction of the synthesized proteins that include additional amino‐acids [2, 3, 4, 5, 6]. It should be noted that the “failure” of the programmed translation termination in the stop codon is not merely a translational error. In fact, several biologically important proteins are synthesized as a result of functional translational read‐through [7, 8, 9, 10]. Indeed, beyond the classical mechanism of stop, some alternative modes of suppression of the translation termination are known: (i) ribosomal frameshifting [11,12], (ii) misreading the termination codon by suppressor tRNAs [13,14], and (iii) stop codon read‐through (SCR) [15, 16, 17]. In the last case, instead of the recognition of the termination codon by the release factor eRF1, a near‐cognate tRNA accommodates in the ribosomal A‐site and a new amino acid is incorporated into the polypeptide chain. In this way, the competition between the release factor eRF1 and a near‐cognate tRNA with the ability to pair 2 of the 3 positions of the stop codon, define the efficiency in the termination process of protein synthesis. The efficiency of this termination process varies between the three stop codons. In fact, it is known that UGA codon has the highest stop codon leakage rate, but also the lowest fidelity; that UAG is the most trusty stop codon, and that UAA has the highest fidelity [4,8,18,19]. Furthermore, the misreading rate can be affected by the nucleotide context around the stop codon [4,17], but also by regulatory elements located long away on the transcript [8,13,16]. Moreover, it also can be induced by pharmacological agents [20, 21, 22, 23]. The mechanisms that operate this regulation are still not well understood and remains elusive. At this point, it would be convenient to define as a basal SCR, those events in which translational read‐through dependent solely on nucleotide context around the stop codon. It have been estimated that the basal stop codon leakage rate is lower than 0.1% [22], but there exist factors that can increase read‐through by several orders of magnitude, resulting in rates higher than 1% [8,22] and suggesting that SCR is a functional recoding mechanism to extend the proteins at the C‐termini [17]. This programmed SCR offers the organisms another way to expand the capacity of genomes, other than splicing.

The rules governing the efficiency of SCR still remain poorly understood. Functional SCR was originally discovered in the bacteriophage Q β [24] and in the tobacco mosaic and barley yellow dwarf viruses [25,26]. More recently, SCR was documented on some few genes in fungi [5,27] and higher eukaryotes, such as β ‐globin gene in rabbits and *syn* and *hdc* genes in *Drosophila melanogaster* [15,28,29], to mention some few examples. However, by the use of different systems biology approaches, in the last years, some hundreds of new SCR events have been identified in several metazoan genomes, suggesting that this is a pervasive mechanism. Among these analyses we can mention the comparative phylogenetic studies [4,30,31], the ribosome profile based approach [32], and a linear regression based model for analysis of stop codon context [33].

The phylogenetic approach applied to twelve *Drosophila* genomes identified more than 280 genes undergoing SCR. Interestingly, one third of these genes contains UGA codon followed by C [4]. Moreover, an improved comparative method performed recently, added more than 50 possible SCR events previously undetected in *D. melanogaster*, and 353 in the genome of *Anopheles gambiae* [31]. Furthermore, the mRNA regions that are being actively translated can be recognized by the ribosome profiling technique [34], which might contribute to identify those sequences occupied by ribosomes downstream of the stop codon. A partial examination of the ribosomal footprint profile from *D. melanogaster* embryos and S2 cell line identified 350 SCR putative events [32], including 43 previously detected by the phylogenetic approach [4]. More recently, Schueren *et al.* have introduced an *in silico* method based on a linear regression analysis between SCR frequencies and their respective sequence context of 15‐nt at stop codon [33]. They used 66 experimentally assessed sequences from human genes [22] as a training set, obtaining a model with the ability to quantify the influence of the stop codon context on the SCR. In this way, they have predicted 57 candidates, 6 of which have already been experimentally confirmed [8,35]. The advantages and weakness of these approaches have been reviewed in [10].

In this work, we expand and combine the last two approaches. First, we examined the ribosome profile of 6739 transcripts from *D. melanogaster* experimental embryos, selecting 1176 SCR events. The SCR frequencies and the associated sequence context at the stop codon were used as a training set for the subsequent regression analysis. The large set of SCR frequencies obtained by the ribosome profiling technique allowed us to formulate more complex models. In this sense, we take into account a context of 60 nucleotides length and a procedure to reduce the number of parameters to be determined in the regression step.

Unlike other models, our modeling approach is applied to each of the three stop codons separately. The results that emerge from our analysis are somewhat surprising. Indeed, our approach reveals that context sequences in transcripts with high rates of SCR associated with stop codons UGA and UAA are quite different.

## RESULTS

**Figure 1:**
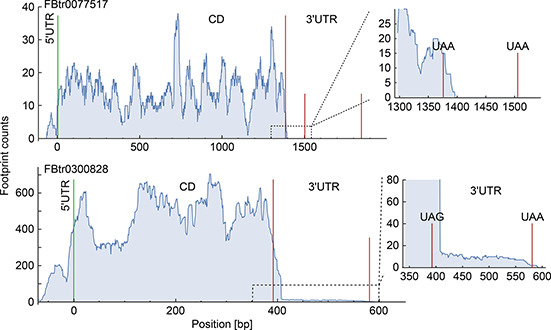
**Examples of ribosomal density profiles with and without SCR.** The upper panel shows the ribosomal profile of the A isoform of the S*nx1* gene (transcript FBtr0077517), which does not present SCR. The green vertical line in the position zero corresponds to the translation start codon, delimiting the CDS of the 5′ UTR end. The red vertical lines indicate the location of the annotated stop codon (UAA) at position 1376 nt, and a posterior stop codon (UAA) located 129 nt later. The 3′ UTR region after the annotated stop codon (inset) shows the absence of ribosomal reads, as canonical translation termination is efficient. The lower panel shows a ribosomal profile with evidence of SCR in the F isoform of the *RpS15Aa* gene (transcript FBtr0300828). This example presents an extension after the annotated stop codon (UAG), located at position 393 nt respect to the start codon. The ribosomal density is extended beyond the first stop codon, reaching the second stop codon (UAA) located 189 nt after it (inset).

After carefully analyzing the ribosome profile of 6739 transcripts expressed during the early embryo stage of *D. melanogaster*, we have identified 1301 cases displaying no null ribosome density beyond the annotated stop codons. Fig.1A shows the ribosome density profile associated with transcript FB0077517 (A isoform of *Snx1* gene), which does not present evidence of SCR. In fact, there is not any complete ribosome footprint read mapped downstream to the annotated stop codon. Moreover, the small tail of 20 nt is associated with the footprints at the stop codon position, where the ribosome is released. On the other hand, when ribosome read‐through the stop codon, the ribosome (density) profile presents a ribosome density between the annotated stop codon and a second stop codon in the 3′UTR. This is the case of the transcript FB0300828 (F isoform of *RpS15Aa* gene) ( Fig.1B). While its density level is lower than the one recorded in the coding region, it corresponds to a substantial number of reads aligned to a transcript portion of 170 pb. In fact, this kind of ribosome density pattern have been considered to constitute a reliable marker of SCR events [31,32]. Some of the SCR events identified in this work have been previously reported. Indeed, 283 candidates have been reported by Jungreis *et al.* [4]; another 307 by Dunn *et al.* [32]; and 486 were annotated in Flybase [36].

**Figure 2:**
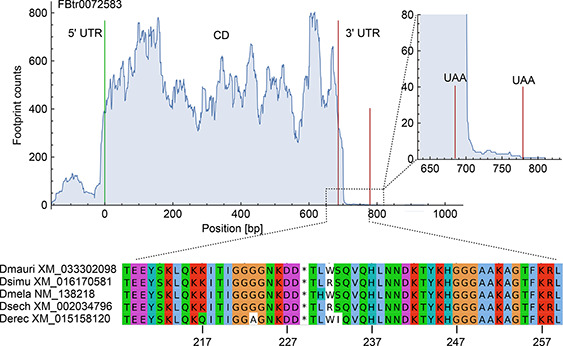
**An example of phylogenetic conservation of a SCR extension.** Ribosomal density profile of the C isoform of the *CG13887* gene (transcript FBtr0072583), with a SCR event (top panel). This 30 amino acid extension shows a high conservation level regarding the extensions that correspond to 4 other species of the same genus (bottom panel).

Thus, we are reporting now 1176 cases of putative SCR events that have not been previously detected. Due that we examined a greater number of ribosome density profiles, using different ribosomal fingerprint alignment methodologies, we found and report now a greater number of events than those reported by Dunn *et al.* [32]. Among others identified in our analysis, we present as an example a simple SCR event in the transcript FBtr0072583 (C isoform of *CG13887* gene) which is not reported in FlyBase ( Fig.2). Multiple alignment of the 30‐residues extensions from *D. mauritiana*, *D. simulans*, *D. sechellia* and *D. erecta* reveals a high local synteny level among these species. Supplementary Figs. S1−S3 present other three examples of single, double, and triple SCR events corresponding to the A isoform of the *ghiberti* gene (FBtr0076462), *CG11070* gene (FBtr0079297) and the isoform A of the *Nurf‐38* gene (FBtr0072343) respectively.

The SCR events reported here have been registered for the three stop codons individually, identifying 306 for UGA, 555 for UAA and 440 for UAG codons. The gene and transcript IDs, as well as several characteristics of these SCR are listed in Supplementary Table S1. The ribosome profiles associated with all these transcripts are deposited in zenodo. Based on the ribosome density profile, we estimated the SCR rate for each of all these events. Because the ribosome covers approximately 30 nt, a second stop codon close to the annotated one can alter the local ribosome density, generating an unreliable estimation of the SCR rate, those cases in which the distance between the annotated stop codon and the next in frame stop codon is less than 18 bp were excluded for further analysis. Taking into account this feature, we selected 238 SCR events for the UGA codon, 447 for UAA and 341 for UAG. Finally, based on this more confident set, we analyzed the distribution for each one of the three stop codons separately.

**Figure 3:**
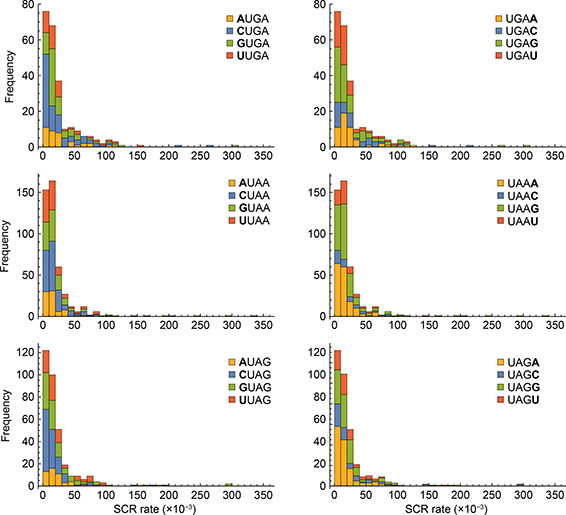
**Frequency of adjacent nucleotides.** Frequency of ribosomal leak rate for the nucleotides left‐ and right‐adjacent to the stop codon (left panels and right panels, respectively). The upper panels show histograms associated with the UGA stop codon, the middle ones show histograms associated with the UAA stop codon, and the lower panels histograms associated with the UAG stop codon. The adjacent nucleotides A, C, G and U are identified by yellow, blue, green and red colors, respectively.

The frequency of the SCR rate for each stop codon shows that for small SCR rates ($<20\times 10^{-3}$ ), most of the events are almost uniformly distributed or with a slight tendency to the UAA and UAG codons; while for higher SCR rates there exists a deviation to the UGA codon (Supplementary Fig. S4). When programmed SCR is associated with high rate, as suggested in [9,37], this result might be indicating that programmed SCR would be encoded in a sequence context using a UGA codon. In this sense, it is interesting to analyze the frequency of the nucleotides distributed around the stop codons. The left panels in Fig.3 show the SCR rate for the nucleotides located upstream from stop codons, while those on the right show the frequency for the nucleotides located downstream. For the case of the UGA codon (top panels), a preference for C and G nucleotides for both, upstream and downstream adjacent positions is evident. This is in agreement with the fact that UGA‐C is one of the less frequent used 4‐nt context in transcripts with efficient termination [4]. The results also show that the fraction of SCR events with higher rate is greater in the case of UGA codon than for the other two stop codons.

**Figure 4:**
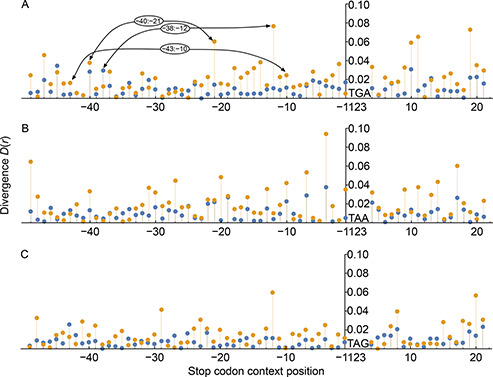
**K‐L divergence values analysis of the context sequence.** *D(r)* of the nucleotide frequency usage in position r for the context sequence associated to each stop codon: UGA (A), UAA (B) and UAG (C), indicated at positions 1‐3. Blue and yellow dots correspond to the divergence values calculated for the transcript sets associated with moderate (TS1) and high (TS2) ribosomal leakage rates, respectively. The nucleotide positions in the context sequence are represented in the horizontal axis, while the divergence in its frequency usage is reflected in the vertical one.

With the aim of find out which nucleotides and positions would play a role in the SCR processes, we performed an alternative statistical analysis, beyond the simple frequency of 4‐nt context. To do that, we computed the frequency of nucleotides usage in a larger stop codon context (SCC) in transcripts presenting SCR events, comparing it with the frequency of nucleotides usage in the same positions, but covering a large control set constituted of transcripts without SCR events. This set will be denoted here by TS0. As a SCC sequence, we considered a region of 49‐nt before the stop codon and 18‐nt after it ( *i*. *e*., 70 nucleotides length). The comparison was made through the Kullback‐Leibler (K‐L) divergence, as indicated in methods. The results show that low values of divergence denote a similar frequency usage of nucleotides in both groups of transcripts, while higher values indicate a preferential usage (bias) linked to SCR events. To go deeply in the analysis, we performed this study for each stop codon separately, using two different sets of transcripts associated with SCR events: (i) transcripts with SCR rate greater than 3 $\times 10^{-4}$ (transcripts set 1, TS1), and (ii) transcripts with SCR rate greater than $20\times 10^{-4}$ (transcripts set 2, TS2). We expected that positions where the K‐L divergence (preference usage) increases by the use of the set with high SCR rate were more relevant to exert influence on the SCR. In Fig.4 we can see the resulting two divergence values for each position of the analyzed SCC. Blue dots correspond to divergence values computed using transcripts that belong to TS1, while yellow dots correspond to the values computed using transcripts associated with higher SCR rate ( *i*. *e*., TS2). From here on, all positions will be referred with regard to the stop codon position. In addition to the K‐L divergence calculation for each position, we also performed a Fisher’s exact test to corroborate the statistical significance of the differential usage of GC nucleotides compared to AU nucleotides, in the different transcript sets. Supplementary Table S2 shows the nucleotide occurrence at each position and the statistical significance of the Fisher’s exact test (p ‐values) of GC and AU occurrence. In the case of the UGA codon ( Fig.4A), we observed that the downstream position adjacent to the stop codon has a preference usage for the nucleotide G. In fact, 34.4% of the transcripts associated with higher SCR rate present this nucleotide, while the nucleotide T is the less frequent (19.3%). Other downstream positions with high divergence values are +9, +10, +11, +19 and +20. Moreover, some of them are even associated to higher divergence values than position +1. For example, the frequency usage of nucleotides A and C on TS0 at position +11 is 27.4% and 23.8%, respectively. However, these nucleotides present very different preferences usage at the same position on TS2, in which the percentages change to 14% and 32.3%, respectively.

Regarding the upstream positions, while −1 present a small divergence value, a significant nucleotide preference is found at position −2, where the frequency usage of nucleotide A reaches 53.8%, and GC nucleotides are significantly less frequent than AU (p ‐values ≥0.05 level). There are also high divergence values at several distal positions, as at −12, −21, −40 and −47. At these positions, the divergence value substantially decreases when the observed nucleotide frequencies in TS1 are used, suggesting that nucleotide at these positions play a role in the SCR rate. In particular, the position with higher divergence is located 12 nt upstream of the stop codon. At this position we found that the frequency usage of nucleotides A, U and G, on TS0, are 30.9%, 16.7% and 30%, respectively. The frequency usage of these nucleotides on TS2 changes to 48.4%, 9.6% and 20.4%, respectively. Additionally, we observed a cluster (between −18 and −12) where divergence values obtained with transcripts belonging to TS2 (yellow dots) are significantly greater than those obtained with transcripts belonging to TS1 (blue dots), suggesting an important role in determining the rate of SCR. Furthermore, our analysis shows that there are more distant positions ( *e*. *g*., −40 and −47), having a remarkable nucleotide preference usage, indicating that they are also important for the SCR process. In particular, we observed a strong usage bias in nucleotides A and U at position −47, which present frequencies of 35.5% and 16.1%, respectively, in TS2. Thus, in contrast to previous studies [4,32], our analysis suggests that distal positions could have also a key role in SCR.

In the case of transcripts with the UAA stop codon ( Fig.4B), the upstream position flanking the stop codon have divergence values higher than in the UGA case, with frequencies of 41.7% (nucleotide C) and 13.4% (nucleotide A) for the most and least used nucleotide at position −1. On the other hand, the most and least used nucleotides at position +1 were G and C, showing frequencies of 41.7% and 13.4%, respectively. We have also observed a high divergence value at position −4, associated with a strong usage bias in nucleotide C, which has a frequency of 48% in transcripts associated with higher SCR rate. Other interesting feature revealed by the divergence analysis of the transcripts with SCR, using UAA as stop codon, is that there is a remarkable nucleotide preference usage at the third position of the codon as it is seen, for example, at positions −1, −4, −7, −10, −13, −16, −19, −31, −40 and −49. All these positions are associated to higher content of GC nucleotides when compared with AU nucleotides. Thus, the GC3 content is significantly higher at the 0.01 level (see p ‐values in Supplementary Table S2). In fact, the GC percentages at these positions are: 69.3, 71.6, 70.0, 72.4, 72.4, 70.9, 70.1, 74.8, 66.1 and 78.7 respectively. These values are much higher than 60.7, which is the average GC content observed at these positions when computed over all transcripts ending in the UAA stop codon. This pattern remarkably differs compared to the cluster found in the UGA case, indicating that mechanisms of SCR in these codons could be not the same. There is a remarkable nucleotide preference usage observed at position −49, the most distal position analyzed in this work. Here, the frequency usage of nucleotide G represents 44.9% of the transcripts in TS2, while the nucleotide A represents only 6.3%. In the case of transcripts with the UAG stop codon, we observed in general lower divergence values than in previous cases, with some exceptions at positions −48, −29, −12, +8, +15, +18 and +20 ( Fig.4C). The position −12 of TS2 transcripts presents significantly higher GC content than control transcripts. Both immediately adjacent positions to the UAG codon does not present any important nucleotide bias respect to TS0.

In addition to correlating the nucleotide composition with SCR rate at individual positions in a large SCC, we also analyzed the probable existence of a correlation between nucleotides at 2211 position pairs in the SCC. As we detailed in methods, we computed the divergence between the joint probability $p_{ij}$ of nucleotides, at the position pair *i* and *j*, and the expected probability assuming statistical independence ( *i*. *e*., $p_{i}\times p_{j}$ ). Large divergence values indicate that nucleotides appear in a concerted manner at given position pairs. The Fig.4A shows some relevant position pairs for the UGA stop codon, which present two nucleotides in a concerted manner even at distal positions, being the pairs −40:−21, −38:−12, −43:−10, −16:−15 and −21:−10, some of the most relevant. As an example, the pairs C:G and G:G appears in 32.2% of the transcripts at positions −40:−21. Perhaps, the most interesting case is represented by the adjacent positions −16:−15, within the cluster indicated in the previous analysis. In this case, the probability of finding the nucleotide pairs C:A or G:G reaches up to 1/3, when the expected value by chance is 1/16.

Another adjacent positions pair that presents nucleotides in a concerted manner are positions 19:20, downstream the stop codon. At these positions, the occurrence of nucleotides C:A and G:C increases up to 30.1% of the cases, almost two‐fold than the expected value assuming independence (17.3%). In the case of UAA stop codon, we found that the nucleotides G:C are over‐represented at positions −21:−4, respectively, with a frequency of 22%. We have not identified statistical dependence between the nucleotides at the third position in different codons, indicating that, in this case, the GC3 content and not one specific pattern rich in GC3, is associated with the high SCR rate. Regarding the UAG stop codon, we observed a significant correlation at three adjacent position pairs upstream the stop codon. In this sense, positions pair −13:−12 evidences a preference for the nucleotide pairs G:G, C:U and G:C, which reaches up to 42.6% of the total cases. At positions −9:−8, the probability to found the nucleotide pairs G:A and A:A increases up to 33%, while at positions −5:−4 the probability to found the nucleotides pairs A:G and G:C is 29.5%.

**Figure 5:**
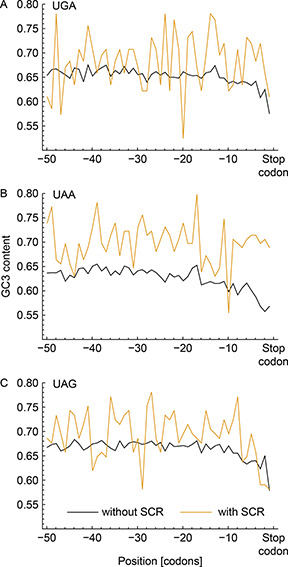
**GC3 content in SCR.** The fraction of G or C nucleotides at the third position in the codons vs codon position for each stop codon: UGA (A), UAA (B) and UAG (C). Yellow lines correspond to the values calculated for the transcript sets associated with high ribosomal leakage rates (TS2), while the black lines correspond to all transcripts with the same stop codon but excluding the transcripts with SCR. The position corresponding to the stop codons was excluded from the analysis.

The results of our divergence analysis, particularly those related to Fig.4B, suggest that the use of codons could play a role in the final stretch of translation. It is well known that the use of codons present bias in different manners, and their frequency usage vary between genes of the same organism [38], and even between the different regions of the same gene [39]. In this sense, some researcher have suggested that codon usage could modulate the speed of protein synthesis [40,41]. Since the synonymous codons are determined mainly by the third nucleotide, it is then useful to evaluate the content of GC3. Fig.5 shows the behavior of the GC3 content in a set of transcripts presenting a high rate of SCR (TS2, yellow lines) and for transcripts that do not present SCR (TS0, black lines). For those transcripts that do not show SCR, a descending ramp in terms of GC3 content in the last 5 codons is evident in comparison with coding senquences ending on UAA presenting SCR ( Fig.5B), where the ramp is not present. We have also detected an ascending GC3‐ramp at the beginning of translation (see Supplementary Fig. S5), that might be related to the known fact that suboptimal codons at the 5' end slowdown translation [42]. Thus, the result exhibited in Fig.5B suggests that SCR in coding senquences ending on UAA can be mediated by the lack of ribosomal pause at the end of the transcript.

**Figure 6:**
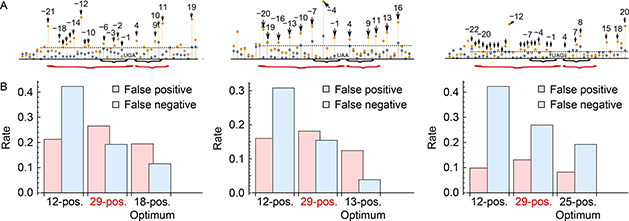
**Predictive models for different size context sequences.** The top panels illustrate the nucleotide positions in the context sequence, used by three different linear models to predict SCR for the stop codon transcripts UGA, UAA, and UAG, respectively. Black braces indicate the 12 positions considered by [33], and the red ones consider an extended pattern including the 29 positions contiguous to the stop codon. Black arrows consider only 18 positions selected due that they have the highest divergence values associated. The bars in the lower panel show the fraction of erroneous predictions: false positives (pink) and false negatives (light blue), for the three models indicated in the upper panel.

Our working hypothesis is that the nucleotide configuration in the SCC can increase the probability of ribosomes interpreting the stop signal in an alternative way, increasing the SCR rate. The divergence study performed above determines which positions within the SCC might be important in the determination of SCR events and their rates. One possible way to corroborate the role of certain positions is through predictive models similar to those implemented by Schueren *et al.* [33], but performing them in a little more sophisticated way. In this sense, we have developed two linear models that differ in the positions incorporated as relevant information. After determining the coefficients of the models (see Methods), we estimated their performance to predict the SCR rate. Then, we compared the predictive performance of a model that takes into account the nucleotide content for the whole SCC, with another model that only takes into account the nucleotide content in a given relevant position. These positions are those indicated by black arrows in the upper panel of Fig.6A. For each case, the model parameters were determined performing a linear regression, using SVD as indicated in the Methods section. Then, the predictive power of the model was evaluated using a particular set of test sequences for each stop codon. These test sequences include transcripts that present SCR, as the annotated ones in the FlyBase database, and that were not used for the determination of the parameters values of the models. Furthermore, the test set includes 50 sequences that do not display SCR for the same stop codon. Although the model predicts the leak rate associated with a given context sequence, the evaluation of its performance was carried out through the calculation of the fraction of false positives and false negatives, and not by the difference between the experimental ribosomal leak rates predicted by the model. Namely, false positives are those sequences that are not associated with SCR, although the model predicts a positive leak rate. On the other hand, false negatives correspond to those sequences that present SCR according to the FlyBase database, but for which the model predicts a negative leak rate. The goal is to obtain models that minimize both types of errors while maximize the correct predictions.

Fig.6A shows the false positive and false negative fractions obtained from three different models developed for transcripts with the UGA stop codon, and using different positions in the context sequence. The first pair of bars corresponds to the model that only takes into account six contiguous positions on both sides of the codon, in a similar way performed by Schueren *et al.* [33]. This model identifies the existence of SCR events in almost 80% of the transcripts that present this phenomenon. However, it has a high fraction of false negatives. This fraction decreases by half when we extend the model to 29 positions. On the other hand, the inclusion of the 29 positions decreases the prediction of the transcripts that present SCR. This fact suggests that the relationship between the number of sequences in the training set and the number of parameters to be determined has a considerable impact on the performance of the modeling. That is, when the number of sequences in the training set is kept constant, to increase the number of positions used in the model does not guarantee a better predictive power. Keeping this in mind, it is interesting to consider evaluating a model that incorporates only the most informative positions, reducing the number of parameters to be determined. In this sense, the model that only uses the 18 optimal positions (black arrows) has lower false positive and negative rates than the other models.

Similarly, Fig.6B shows the false positive and false negative fractions obtained from three models corresponding to the UAA stop codon, which use different positions in the context sequence. The first pair of bars corresponds to the model inspired by Schueren *et al.* [33], which only takes into account 6 contiguous positions on both sides of the UAA codon. This model identifies the presence of SCR events in almost 80% of the candidate transcripts. However, it has a high fraction of false negatives. As in the case of the UGA codon, this fraction decreases by half when we extend the model to 29 positions. On the other hand, the inclusion of the 29 positions increases the prediction of transcripts that present SCR by 0.2%, although the proportion of this increment is lower than the previous case. Again, this indicates that the relationship between the number of sequences in the training set and the number of parameters to be determined ( *i*. *e*., the number of positions used) alters the performance of the model, and not guarantee a greater predictive power. In this case, with the aim of reduce the number of parameters to be determined, the model was evaluated incorporating only the 13 most informative positions (black arrows in the figure). This model shows false positive and false negative rates markedly lower than the other ones, being both rates significantly lower than the 18 position optimal UGA codon model. Furthermore, this false negative rate is the lowest when the optimal positions evaluated for each stop codon are compared.

Finally, Fig.6C shows the false positive and false negative fractions obtained from four different models for the UAG stop codon, which use different positions in the context sequence. The first pair of bars corresponds to the model that only takes into account six contiguous positions on both sides of the UAG stop codon. This model identifies the presence of SCR events in almost 80% of the candidate transcripts. However, it predicts a high fraction of false negatives. This fraction decreases considerably when we extend the model to 29 positions. On the other hand, the inclusion of the 29 positions increases by 0.15% the prediction of the transcripts that present SCR, although this increment proportion is the smallest observed for the three cases. The reduction in false negatives linked to a slight increase in false positives observed here, would indicate that the relationship between the number of sequences in the training set and the number of parameters to be determined favorably alters the performance of the model. However, the evaluation of the model, when only the most informative positions are incorporated, shows a better predictive power than the other models, by reducing the number of parameters to be determined. In this case, the model that only uses the optimal 25 positions (black arrows in Fig.6), shows an appreciably lower rate of false positives and false negatives than the other models. It is even observed that the false positive rate is the lowest compared to all the applied models; while the false negative rate is the highest among the three models that use the optimal positions in the context of the stop codon.

## DISCUSSION AND CONCLUSION

Based on ribosomal leak rate estimated from Ribo‐seq data, we determined the ribosomal density profiles for the 6739 observed transcripts of *Drosophila melanogaster*. The examination of 3′UTR regions of these profiles allowed us to identify 1176 putative SCR events, with an incidence of 23%, 33%, and 44% for the UGA, UAG, and UAA stop codons, respectively. According with previous findings [8,18,31], we also observed that SCR events associated with the UGA codon have a higher ribosomal density, indicating a greater ribosome leak rate, particularly with UGA‐C. The fact that a high rate of ribosomal leakage is associated with the presence of a pyrimidine at position +4 might be in agreement with previous findings, since electron cryo‐microscopy data suggest that the compaction of mRNA by eRF1, which is necessary for codon recognition stop, is facilitated when +4 corresponds to a purine and not to a pyrimidine [1]. The correlation between this increment in the ribosomal density and the nucleotide usage frequency, immediately after the stop codon, may indicate that SCR events are caused by an implicit mechanism in the nucleotide sequence rather than translational decoding errors [17,32,43,44]. In order to identify patterns that can predict SCR events, in the present study, we evaluated the influence of the nucleotides in a large stop codon context region. This analysis was performed by the Kullback‐Leibler measure of divergence, which indicates the presence of tendencies in the use of nucleotides at each position. We found high divergence values at various upstream distal positions, many of them specific to each stop codon. For example, the UGA codon shows high divergence at positions −2 and −12, using mainly the nucleotide A (53.8% and 48.4% respectively). The positions with a strong tendency to the use of a certain nucleotide, may indicate a marked influence on the occurrence of programmed SCR events. Furthermore, our study has demonstrated a high frequency usage of nucleotides G or C (around 75%) in the third base of several codons of transcripts with the UAA stop codon. This is higher than the proportion previously observed in the same positions of the control group (60%), and the frequency expected by a uniform use of codons (50%). This notable bias differs widely from that observed in UGA, indicating that the SCR mechanisms would be operating under patterns of differential use of specific nucleotides at distal positions for each type of stop codon, something that was not contemplated in previous studies [4,17,32]. In the case of transcripts with the UAG stop codon, the divergence in nucleotide usage was generally smaller than the ones observed for UAA and UGA, and did not show biases in adjacent positions. Our analysis of nucleotide pair divergences indicates the existence of very few pairs with high divergence, and that these are not located in a coordinated manner necessary to support the typical base paired hairpin structures. Therefore, they do not seem to support the hypothesis that secondary structures have a role in SCR.

We are proposing now a set of regression models which differ on the size of the SCC used to make the prediction. Indeed, the aim was to compare the influence of such nucleotides on the leak rate of each stop codon independently, in order to corroborate the role of the most relevant positions identified by the K‐L divergence analysis. The model with 6+6 positions contiguous to each stop codon identifies SCR events in 80% of the transcripts evaluated, but presents a high fraction of false negatives for all cases. The model that uses a SCC with 29 positions contiguous to the stop codon considerably reduces the number of false negatives obtained by the previous model, but increases the fraction of false positives. This shows that the size of the context sequences used as a function of a fixed number of training sequences does not guarantee a reliable predictive power. On the other hand, the model that includes only the positions with a high divergence value associated with the context of each stop codon with SCR (contiguous or not), was effective by significantly reducing the rate of false positives and negatives with respect to the two other models. Finally, the use of the most informative positions regarding the level of divergence within a context sequence constitutes a novel and useful criterion for the development of computational tools such as the one presented here.

One of our most interesting finding is that, except for the case of transcripts with UAA stop codon that present high rate of SCR, there exists a lower GC3 content at the 5′ end in almost all transcripts of *D. melanogaster*. The presence of this codon bias could be related with the ribosomal pause needed for the compaction of mRNA and the posterior stop codon recognition [1]. Taking this into account, we hypothesized that high GC3 content in the last codons associated with coding sequence ending on UAA could be led to a stop codon recognition failure with the consequent ribosomal leakage. This hypothesis might be contrasted by means of suitable molecular biology experiments.

On the basis of the analysis detailed above, we would wish to propose that divergence analysis could be used as a criterion to select the most informative positions in the modeling. Moreover, our results indicate that the rate at which SCR events occur could be regulated by a context greater than those proposed by previous studies [4,33]. Furthermore, it is important to clarify the relationship between the number of parameters and the number of sequences. Although it is clear that the larger the size of the training set, the better the parameter fitting of the model; this is not the case in relation to the number of parameters.

In conclusion, this extensive and deep study of the existing relationship between most nucleotide positions and ribosomal leakage rate allows not only the recognition of a larger set of genes that might undergo SCR process, but also reveals implicit regulatory mechanisms at the nucleotide sequence, which might regulate translational ending, having broad and relevant biological implications across several kingdom.

## METHODS

**Figure 7:**
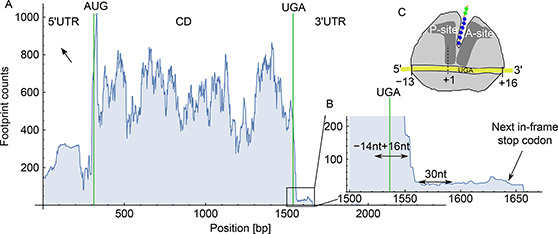
**Ribosomal leak rate estimation.** Panel A represents a typical ribosomal density profile, distinguishing the 5′ and 3′UTR ends of the coding region delimited by the AUG start and UGA stop codons (vertical green lines). An enlargement of the stop codon region and the contiguous C‐terminal extension is shown in (B). Here, the ribosomal density accumulated during the 30 nt associated with these regions: δ_CD_ and δ_ext_ is shown (light blue). Panel C shows the location of the ribosome A‐site relative to the UGA stop codon.

We used the raw data of ribosome footprint profiles from *Drosophila* embryos (0−2 h) obtained by Dunn *et al.* [32] (available for download at NCBI GEO, accession #GSE49197). At first, ribosome footprints reads were trimmed to remove the adapter sequence using the Cutadapt software [45], giving as result that reads shorter than 25 nt or with low‐quality were discarded. Further, we used the Bowtie 2 software [46] for also discard the reads that align to ribosomal sequences. Unlike to the analysis performed by Dunn *et al.* [32], and in order to align the remaining reads to the FlyBase *Drosophila* genome (version r6.03), we used the Tophat software [47], which takes into account also splice junctions. The resulting SAM files were processed with SAMtools [48] to compute the ribosome density profile (total number of ribosome protected footprint fragments aligning to each nucleotide position) of all transcripts ( Fig.7A).

The SCR rate, denoted here by ρ , which is associated with each annotated stop codon, was computed by dividing the cumulative ribosome density associated with a C‐terminal extension region, $\delta_{ext}$ , by cumulative ribosome density associated with the coding region immediately upstream of the stop codon, $\delta_{CD}$ ,

(1) $$\rho =\frac{\delta_{ext}}{\delta_{CD}},$$

$\delta_{CD}$ was computed taking into account that ribosomes protect fragments of 28 to 29‐nt‐long, and that the P‐site of the ribosome is located at position 13, as shown in Fig.7B. Thus, we considered the region ranging the 14 nucleotides preceding the stop codons of the coding region, and the 16 nucleotides following the stop codons ( Fig.7B, C). For computing the cumulative ribosome density associated with the extension, $\delta_{ext}$ , we considered a 30‐nt‐long region that range 29 to 58 nucleotides downstream of the stop codon, as indicated in Fig.7B. In this way, we decreased the chance to count ribosome protected fragments from the end of the coding region as part of an extension, decreasing so the number of false positive SCR cases. As putative cases of SCR, we selected those transcripts that satisfy two criteria: (i) ribosome‐protected footprint fragments must cover at least 90% of the extended region with a minimum of 2 reads. (ii) SCR rate is greater than 0.005.

After selecting all compliant transcripts, each associated ribosome density profiles was visually inspected to discard artifacts and to choose only the correct isoform. Then, we discarded those transcripts with a second stop codon close to the annotate one, and selected 238 SCR transcripts for the UGA codon, 447 for UAA and 341 for UAG stop codon. In this way, a number of 1026 transcripts with at least one SCR event were selected. All these positive cases of SCR were used to build three different predictive models, each one for every stop codon.

As there is not information about the size and positions of the stop codon context (SCC) that can exert influence on SCR events *a priori*, Schueren *et al.* [33] considered a SCC of 15‐nt long. That modeling was restricted to consider a small SCC because the available training set consisted of only 66 sequences. The large size of the training set available here allows us to explore more complex modeling ( *i*. *e*., with large number of entries). In this sense, we proposed a model in which the SCR rate depends on the nucleotide $x_{i}$ at position i in a SCC of 70 nucleotide length, 49‐nt before the stop codon and 18‐nt after it. Thus, our model for the rate y can be written in the following manner

(2) $$\begin{array}{l} y=a_{0}+\sum_{i}^{*}b_{i}x_{i}, \end{array}$$

where the asterisks in the summation indicate that the index runs over the selected position and positions pairs. The terms $a_{O}$ , $b_{i}$ and $x_{i}$ are the parameters of the model, which must be determined. As the number of parameters to be determined would be similar to the size of our training set, this can lead to a poor performance of the model. To overcome this difficulty, our idea is to determine which positions of the SCC are more likely to exert influence on SCR. Our working hypothesis is that those positions that present a bias in the nucleotide usage are more likely to affect the SCR rate; reaching in consequence the more informative for the modeling. In this sense, from all sequences of our training set we computed the Kullback‐Leibler divergence [49] D(r) at position r of the SCC. This measure, defined as $D(r)=\sum_{i}p_{i}(r)\log(p_{i}(r)/p_{i}^{*})$ , quantify how the frequency usage of nucleotide i in the position r , denoted by $p_{i}(r)$ , is different from the frequency usage of this nucleotide over the SCC of reference transcripts, denoted by $p_{i}^{*}$ . Moreover, in order to also include in the modeling information from position pairs, we also computed the following Kullback‐Leibler divergence

$$D(r,s)=\sum_{(i,j)pairs}p_{i,j}(r,s)\log\left(\frac{p_{i,j}(r,s)}{p_{i}(r)p_{j}(s)}\right),$$

where $p_{i,j}(r,s)$ is the frequency of the nucleotides i and j at the positions r and s respectively, while $p_{i}(r)$ is the frequency of the nucleotide i at the position r . This measure quantifies how the frequency of nucleotide usage at two different positions is statistically correlated in the sequences belonging to the training set. After evaluating these divergences, we selected as variables for our model the most informative individual positions. As this step was performed independently for each training set corresponding to one of three stop codon signals, the resulting nucleotide positions are not necessarily the same for the three training sets.

**Figure 8:**
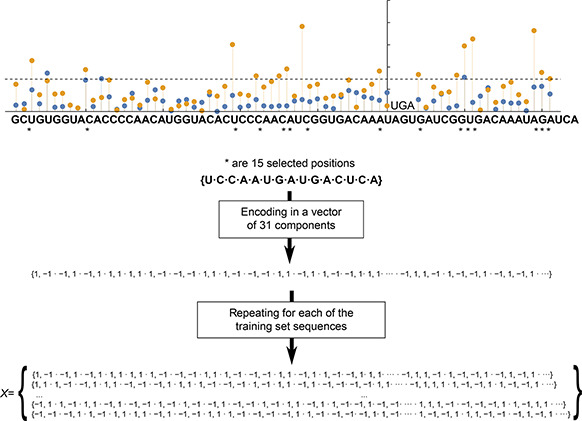
**Numerical coding of the nucleotide sequence context.** This illustrative diagram represents how the context sequences are encoded numerically to feed the linear model. The * indicate the selected nucleotide positions. Each nucleotide is encoded by a pair of 1 or −1. The procedure is performed for all context sequences and the matrix *X* is constructed.

The relevant single nucleotides corresponding to individual positions of the SCC can be represented by two elements: (A→ {1,1} , C→ {−1,1} , U→ {1,−1} and G→ {−1,−1} ). The encoding procedure is represented in the Fig.8. In order to simplify the notation for the parameter estimation procedure, we noticed that the Eq. (2) can be rewritten as $y=w_{N}\cdot v$ , where w is N ‐dimensional vector that include all coefficients to be determined ( *i*. *e*., $w=(a_{0},b_{i})$ , and v is an extended version of the feature vector that encode the relevant information of the nucleotide sequence. The training sets consist of M pairs of input‐output, represented by D={X,y} ; where X is an N×M matrix of all sequences in the training set. In this way, the columns of matrix X , correspond to the M sequences, while the rows correspond to the informative positions. The vector y corresponds to M values of the SCR rate. For the estimation of the model coefficients, we have used the least‐squares regression based on the singular‐value decomposition (SVD) of matrix $X^{T}$ , where superscript *T* denotes the transpose matrix ( *i*. *e*., $X^{T}={U\cdot S\cdot V}^{T}$ ); and where U is a unitary M×N matrix of left eigenvectors, S is a diagonal N×N matrix containing the eigenvalues $\left\{s_{1},\ldots ,s_{N}\right\}$ , and V is a unitary N×N matrix of right eigenvectors. Thus, the solution with the smallest $L_{2}$ norm is given by $w=y\cdot U\cdot diag(s_{j}^{-1})\cdot V^{T}$ , and w⋅v corresponds to the SCR rate predicted by the model for a sequence feature vector v .

## DATA AVAILABILITY

Plots of the ribosomal density profile associated with all transcripts with SCR event can be found at Zenodo: Distal nucleotides affect the rate of stop codon read‐through, zenodo.4633888. Data are available under the terms of the Creative Commons Attribution 4.0.

## SUPPLEMENTARY MATERIALS

The supplementary materials can be found online with this article at https://doi.org/10.15302/J‐QB‐022‐0298.

## AUTHOR CONTRIBUTIONS

LIE constructs ribosomal density profiles, participated in the statistical analysis and revised the manuscript. AMA carried out statistical analyses and modeling. JR conceived the study, coordinated the study and revised the manuscript. LD performs the divergence analysis, conceived the study and coordinated the study and wrote the draft of the manuscript. All authors read and approved the final manuscript.

## COMPLIANCE WITH ETHICS GUIDELINES

The authors Luciana I. Escobar, Andres M. Alonso, Jorge R. Ronderos and Luis Diambra declare that they have no conflict of interest or financial conflicts to disclose.

This article does not contain any studies with human or animal subjects performed by any of the authors.

## Supporting information

Supplementary Information

Supplementary Information

Supplementary Information
