# Supplementary material for: Distal nucleotides affect the rate of stop codon read‐through
Source: Quant Biol. 2023 Mar 1;11(1):44–58. doi: 10.15302/J-QB-022-0298 (PMC12807244; doi:10.15302/J-QB-022-0298)
Supplement: Supplementary file 1 — Supplementary Information [file QUB2-11-44-s002.pdf]

Distal nucleotides affect the rate of stop codon read-through

Supplementary Material

Supplementary Table Legends

Table S1: The gene and transcript IDs, extension and SCR rate, and features, of the SCR events reported here are listed. They are listed separately for each stop codon: 306 for TGA, 555 for TAA, and 440 for TAG codons. Table available at zenodo website (`record/4633888`).

Table S2: The frequency nucleotide usage in the context sequence for each stop codon. They are listed separately for each stop codon (TGA, TAA and TAG). Table available at zenodo website (`record/4633888`).

Supplementary Figures

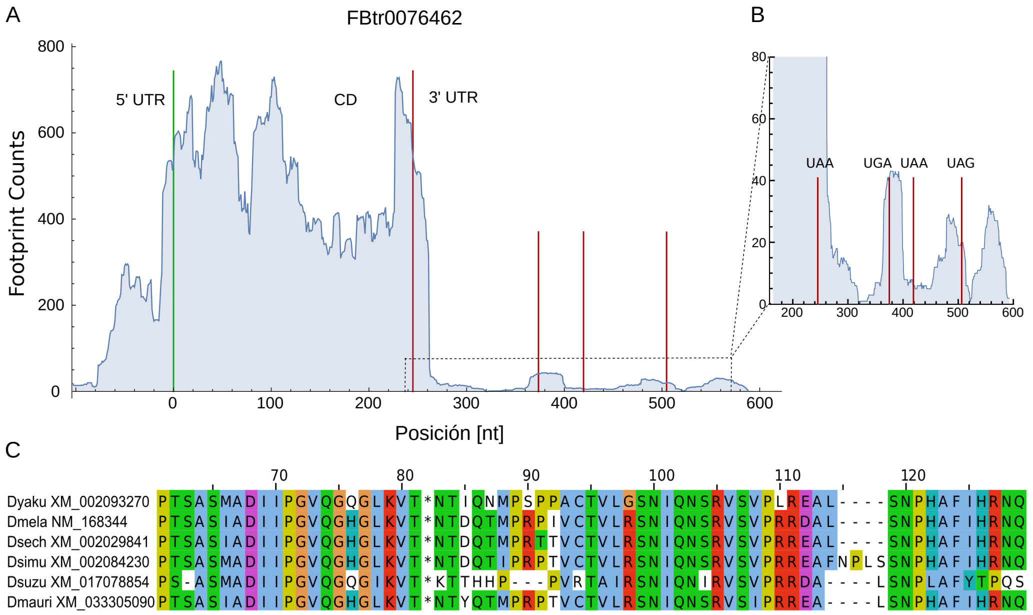

Figure 1: **Phylogenetic conservation of an SCR extension.** In the top panel, the ribosomal density profile of the A isoform of the *ghiberti* gene (transcript FBtr0076462), with a triple SCR event is shown. The *ghi* gene encodes for a homologous protein to the small regulatory subunit of the serine palmitoyl transferase in mammals. Mutations in *ghi* affect the first steps of sphingolipid biosynthesis and alter meiotic cytokinesis in males. This extension presents 43 amino acids between the first and second stop codons. In this case is evident that the ribosomal density peaks at the extension stop codons are similar to the density peaks at the annotated stop codons. The synthenia analysis carried out shows significant similarity when compared with extensions corresponding to five other species of the same genus, indicating a high level of conservation (bottom panel).

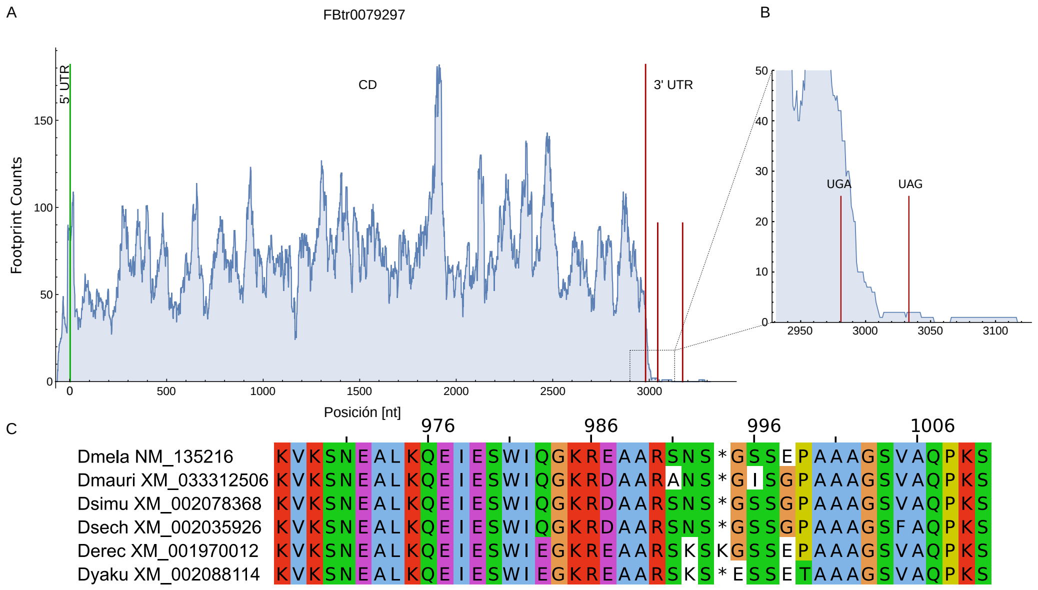

Figure 2: **Phylogenetic conservation of an SCR extension.** Ribosomal density profile of the transcript FBtr0079297 (*CG11070* gene), with a SCR event (top panel). This gene encodes the ubiquitin conjugation factor E4-A.The peptide extension found in this transcript is 16 amino acids longer (GSSEPAAAGSVAQPKS), and it is not currently annotated in FlyBase. When the synthenia analysis is performed, a high conservation level with the extensions corresponding to 5 other species of the same genus is also evident (bottom panel). Note that the homologous sequence corresponding to *D. erecta* has a lysine residue (K) instead of the stop codon, being incorporated into the coding region of the *GG10427* gene; and suggesting that the extension has a biological function.

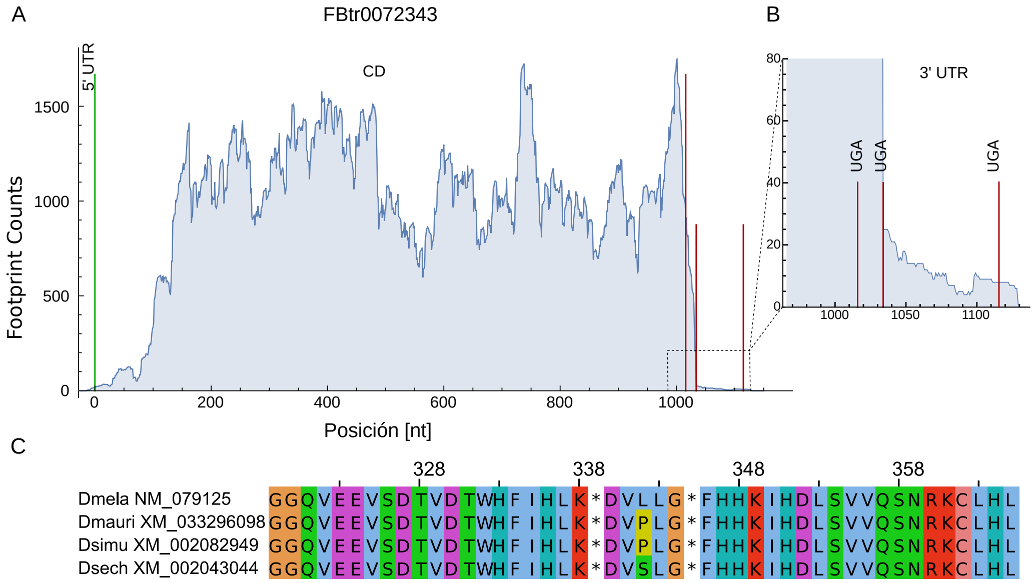

Figure 3: **Phylogenetic conservation of an SCR extension.** Ribosomal density profile of the transcript FBtr0072343 (*Nurf-38* gene), with a double SCR event (top panel). This gene encodes a 38 kD pyrophosphatase, which catalyzes ATP-dependent nucleosome gliding, facilitating chromatin transcription. This 25 amino acids extension shows a high conservation level in four species of the *Drosophila* genus, as shown in the multiple alignment (bottom panel).

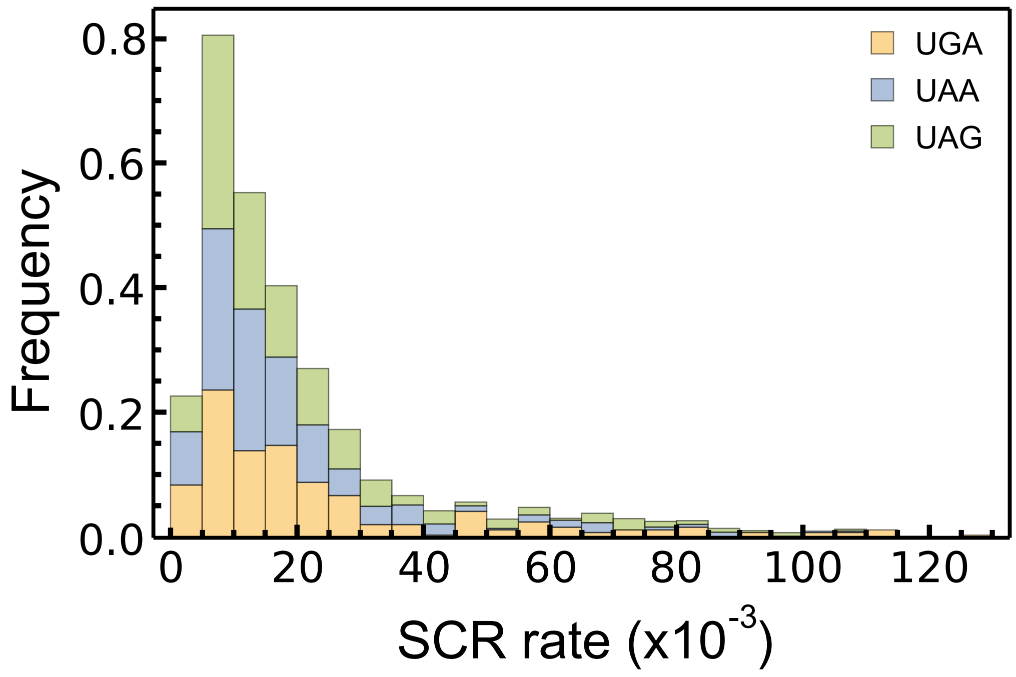

Figure 4: **SCR estimation by stop codon leakage frequency.** Estimation of the LCP rate using frequency histograms for each stop codon (TGA, TAA, TAG), as a function of the leak rate. The stop codons are represented by the segments in yellow, blue and green colors, respectively.

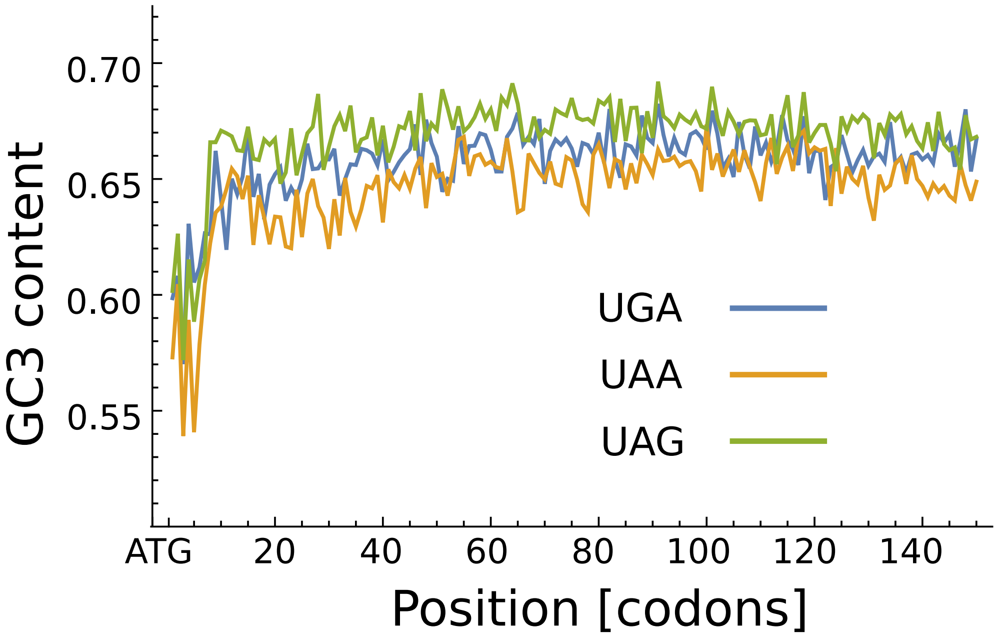

Figure 5: **GC3 content in the 5' end.** The fraction of G or C nucleotides at the third position in the codons *vs* codon position for each stop codon: UGA (Blue line), UAA (Yellow line) and UAG (Greem Line). The values were calculated using all transcripts with the same stop codon. The position corresponding to the AUG codon was excluded from the analysis.
